# Utility of Corrected QT Interval in Orthostatic Intolerance

**DOI:** 10.1371/journal.pone.0106417

**Published:** 2014-09-02

**Authors:** Jung Bin Kim, Soonwoong Hong, Jin-Woo Park, Dong-Hyuk Cho, Ki-Jong Park, Byung-Jo Kim

**Affiliations:** 1 Department of Neurology, Korea University Medical Center, Korea University College of Medicine, Seoul, Korea; 2 Department of Cardiology, Korea University Medical Center, Korea University College of Medicine, Seoul, Korea; 3 Department of Neurology, Kyungsang University Medical Center, Jinjoo, Korea; University of Texas Health Science Center at San Antonio, Research Imaging Institute, United States of America

## Abstract

We performed this study to determine whether electrocardiographic corrected QT (QTc) interval predicts alterations in sympathovagal balance during orthostatic intolerance (OI). We reviewed 1,368 patients presenting with symptoms suggestive of OI who underwent electrocardiography and composite autonomic function tests (AFTs). Patients with a positive response to the head-up tilt test were classified into orthostatic hypotension (OH), neurocardiogenic syncope (NCS), or postural orthostatic tachycardia syndrome (POTS) groups. A total of 275 patients (159 OH, 54 NCS, and 62 POTS) were included in the final analysis. Between-group comparisons of OI symptom grade, QTc interval, QTc dispersion, and each AFT measure were performed. QTc interval and dispersion were correlated with AFT measures. OH Patients had the most severe OI symptom grade and NCS patients the mildest. Patients with OH showed the longest QTc interval (448.8±33.6 msec), QTc dispersion (59.5±30.3 msec) and the lowest values in heart rate response to deep breathing (HRDB) (10.3±6.0 beats/min) and Valsalva ratio (1.3±0.2). Patients with POTS showed the shortest QTc interval (421.7±28.6 msec), the highest HRDB values (24.5±9.2 beats/min), Valsalva ratio (1.8±0.3), and proximal and distal leg sweat volumes in the quantitative sudomotor axon reflex test. QTc interval correlated negatively with HRDB (*r* = −0.443, *p*<0.001) and Valsalva ratio (*r* = −0.425, *p*<0.001). We found negative correlations between QTc interval and AFT values representing cardiovagal function in patients with OI. Our findings suggest that prolonged QTc interval may be considered to be a biomarker for detecting alterations in sympathovagal balance, especially cardiovagal dysfunction in OH.

## Introduction

Orthostatic intolerance (OI) is a syndrome characterized by lightheadedness, fatigue, blurred vision, and loss of consciousness after standing up that is relieved by assuming a sitting or supine posture [1], [2]. Disorders associated with OI are categorized into orthostatic hypotension (OH), neurocardiogenic syncope (NCS), and postural orthostatic tachycardia syndrome (POTS). Distinct abnormal patterns in the autonomic nervous system are the pathogenesis of these three different disorders [3]–[5]. Both parasympathetic and sympathetic dysfunction has been observed in OH [6]. In NCS, decreases in the low-frequency power of heart rate variability have been observed, suggesting a decline in sympathetic activity at the time of syncope [7]. A relative increase in sympathetic activity has been suggested as the mechanism underlying POTS [8]. Thus, disturbances of sympathovagal balance cause OI, which can differentially present as OH, NCS, or POTS.

The QTc interval is an electrocardiographic (ECG) measure of the time between ventricular depolarization and repolarization. Since the QTc interval can be influenced by changes in sympathovagal autonomic modulation [9], it has been used to evaluate autonomic sympathovagal balance in patients with various disorders, as well as in normal subjects [10], [11]. In addition, QTc dispersion, a measure of QTc interval variability, reflects the heterogeneity of ventricular repolarization duration [11]. Increased QTc interval and dispersion, known to provide an electrophysiological substrate for fatal arrhythmia, has been reported in patients with autonomic nervous system disorders such as multiple system atrophy, primary autonomic failure, diabetic autonomic neuropathy, and myotonic dystrophy type 1 [12]–[16]. To the best of our knowledge, few studies have compared sympathovagal balance using the QTc interval in the assessment of disorders associated with OI [17]. In addition, most of the previous studies using autonomic function tests (AFTs) have focused on autonomic dysfunction in a specific disorder without comparing different patterns of autonomic dysfunction between disorders associated with OI. Based on previous findings indicating that distinct patterns of sympathovagal imbalance underlie the characteristics of OH, NCS, and POTS [5], we hypothesized that combined AFT-ECG analysis might provide additional information accounting for differences in the pathophysiological mechanisms of OH, NCS, and POTS.

Thus, in this study we compared QTc interval and dispersion in patients with OH, NCS, and POTS to investigate differences in sympathovagal balance. QTc interval and dispersion were also correlated with AFT measurements to determine whether QTc interval is an applicable biomarker differentiating patterns of altered sympathovagal balance between OH, NCS, and POTS.

## Materials and Methods

### Subjects

We reviewed the medical records of 1368 patients with symptoms of OI who underwent composite AFTs and ECG at a university-affiliated neurology clinic from January 2011 to December 2012. Inclusion criteria were: (1) symptoms indicating OI; (2) positive response to head-up tilt test (HUT); (3) no history of seizure and cataplexy; (4) no history of developmental abnormalities; (5) no proven structural cardiac diseases based on ECG or echocardiogram; (6) no history of significant head injury, alcohol, psychotropic drug abuse, or psychiatric disorders. Asymptomatic stroke and transient ischemic attack including vertebro-basilar insufficiency were also considered comorbid diseases. Demographic and clinical data including age, gender, and comorbid chronic diseases were obtained through medical record review. Symptoms of OI in all participants were classified (using a scale of I to IV) based on the frequency and severity of symptoms [18]; higher grade indicated more frequent and severe OI symptoms.

### Ethics Statement

All participants gave written informed consent before study inclusion. All procedures were in accordance with the Declaration of Helsinki and approved by the Korea University Medical Center Institutional Review Board (IRB NO. ED13036).

### ECG measurements

ECG measurements were performed before AFTs. Measurement of the QTc interval was taken from a standard 12-lead ECG recorded at 25 mm/sec at rest. The QT and RR intervals were measured manually using calipers by one cardiologist (D.H.C.) who was blinded to the diagnosis and other baseline information. The QT interval was defined as the time interval between the onset of the QRS complex to the end of the T wave. The end of T wave was defined as the offset point to isoelectric line using threshold methods [19]. The measured QT interval was then corrected for heart rate (HR) according to Bazzet's formula (QTc interval = QT interval/√RR interval), yielding the QTc interval [11]. The QTc dispersion was defined as the difference between the maximum and minimum QTc interval in the ECG leads [20].

### Autonomic function tests

All participants taking medications affecting autonomic function were asked to discontinue their medicine for at least 24 hours prior to their AFTs. To minimize the effects of confounders, performance of the AFTs was controlled and regulated by standard of electrodiagnostic laboratory environment [21]. Tests were performed in the following sequence: 1) quantitative sudomotor axon reflex test (QSART), 2) heart rate response to deep breathing (HRDB), 3) Valsalva ratio, and 4) HUT.

The QSART, performed with Q-Sweat (WR Medical Electronics, MN, USA), as an index of sympathetic postganglionic sudomotor function [22]. The stimulus consisted of 10% iontophoresed acetylcholine with a constant current generator at 2 mA for 5 minutes. Sweat volumes were recorded in the central compartment of a multicompartmental sweat cell from the following 4 sites: proximal forearm (25% of the distance from the ulnar epicondyle to the pisiform bone), distal forearm (75% of the distance from the ulnar epicondyle to the pisiform bone), proximal leg (5 cm distal to the fibular head), and distal leg (5 cm proximal to the medial malleolus) [22]. The QSART result was considered abnormal if sweat volume was decreased when compared to age- and gender-specific reference values [23].

The HRDB and Valsalva ratio, which reflect cardiovagal function, were measured using a conventional Nicolet Viking IV (Nicolet Biomedical, Madison, WI, USA) according to a previously described method [22]. During deep breathing (6 breaths/minute), the range of HR response to forced respiratory sinus arrhythmia was obtained. HRDB was calculated by subtracting the minimum HR during expiration from the maximum HR during inspiration for each cycle of 6 breaths. For the Valsalva ratio, rested and recumbent subjects blew through a mouthpiece attached to a manometer and maintained a pressure of 40 mmHg for 15 seconds. The Valsalva ratio was calculated as the maximal R-R interval divided by minimal R-R interval. The results were also compared with age-specific reference values [22].

For the HUT test, blood pressure (BP) was taken using a sphygmomanometer cuff over the brachial artery. Systolic and diastolic BP were displayed on a monitor console. Simultaneous HR recording was also performed. After a 20-minute rest in the supine position and measurement of baseline BP and HR, each subject was positioned at an angle of 70 degrees on a standard electrically driven tilt table with a footboard for up to 30 minutes or until symptoms occurred. Serial measurement of BP and HR was performed every minute during HUT. Pharmacologic provocation was not performed [24]. Based on the response pattern to HUT, patients were classified as OH, NCS, or POTS. Patients were classified as OH if a reduction of systolic or diastolic BP of at least 20 mmHg or of at least 10 mmHg, respectively, occurred within 3 minutes after standing up following HUT [25]. Patients were classified as NCS if they reported spontaneous syncope that was associated with hypotension, bradycardia, or both [3], [4], [26]. Patients who displayed HR increases of more than 30 beats per minute or a maximum HR of 120 beats per minute within the first 10 minutes without evidence of OH were classified as POTS [27]. As soon as symptoms occurred, patients were returned quickly to the supine position.

### Statistical analysis

Differences of demographics, symptom grade of OI, QTc interval, QTc dispersion, and measurements of AFTs among the groups were analyzed using analysis of variance (ANOVA) with Bonferroni post-hoc tests. A chi-square test examining the proportion of patients with abnormal results in each AFT was also performed. Additionally, an analysis of covariance (ANCOVA) with age and gender as covariates was performed to control for possible confounding effects on the dependent measures. Pearson's correlations were performed to describe the relationships between QTc interval, QTc dispersion, clinical variables, and AFTs. Partial correlation analyses were then performed between the QTc interval and AFT measurements while controlling for the effects of significant clinical variables identified in the Pearson's correlation analysis. Alpha was set at *p*<0.05. Statistical analyses were performed using SPSS, version 19.0 (IBM. Armonk, New York).

## Results

### Clinical characteristics

A total of 275 patients (159 OH, 54 NCS, and 62 POTS) were included in this analysis. Clinical characteristics and OI symptom grade are summarized in Table 1. There were no gender differences between groups (*p* = 0.09); however, OH patients were older (*p<*0.001) and had a higher proportion of comorbid diabetes, hypertension, Parkinson's disease, and stroke (all *p*<0.05) than NCS or POTS patients. POTS patients were younger than OH or NCS patients (*p*<0.001). POTS patients had a lower proportion of comorbid diabetes, Parkinson's disease, and stroke (all *p*<0.05) than OH patients. Patients with OH reported the most severe OI symptoms, whereas, patients with NCS reported the mildest (*p*<0.001).

**Table 1:** Demographic data and clinical measurements in patients with orthostatic intolerance.

	OH (n = 159)	NCS (n = 54)	POTS (n = 62)	*p*
**Clinical features**				
Age (years)	67.1±12.3 (19 to 88)	50.1±22.4 (14 to 87)	32.0±14.7 (11 to 72)	<0.001
Gender, male N (%)	102 (64.1)	26 (48.1)	34 (54.8)	0.090*
Comorbid diseases				
Diabetes, N (%)	47 (29.6)	9 (16.7)	4 (6.4)	0.001*
Hypertension, N (%)	88 (55.3)	19 (35.2)	6 (10.0)	<0.001*
Parkinson's disease, N (%)	36 (22.6)	1 (1.8)	2 (3.2)	<0.001*
Multiple system atrophy, N (%)	12 (7.5)	0 (0)	1 (1.6)	0.033*
Alzheimer's disease, N (%)	3 (1.9)	2 (3.7)	0 (0)	0.328*
Cerebrovascular disease, N (%)	57 (35.8)	9 (1.7)	7 (11.3)	<0.001*
Symptom grade of OI	3.7±0.5	1.7±0.8	2.9±0.9	<0.001*
**QTc interval (msec)**	448.8±33.6	429.1±24.6	421.7±28.6	<0.001
**QTc dispersion (msec)**	65.1±32.2	49.0±23.2	53.9±20.7	0.021
**AFTs**				
HRDB (beats/min)	10.3±6.0	15.4±9.1	24.5±9.2	<0.001
HRDB abnormality, N (%)	47 (29.6)	8 (14.8)	4 (6.4)	<0.001*
Valsalva ratio	1.3±0.2	1.5±0.2	1.8±0.3	<0.001
Valsalva ratio abnormality, N (%)	51 (32.1)	6 (11.1)	4 (6.4)	<0.001*
QSART (µL)				
Proximal forearm	0.66±0.58	0.48±0.34	0.69±0.60	0.288
Distal forearm	1.26±0.72	1.00±0.56	1.15±0.82	0.329
Proximal leg	0.70±0.60	0.64±0.48	1.21±0.97	0.001
Distal leg	0.56±0.59	0.75±0.59	1.19±0.85	<0.001
QSART abnormality, N (%)	45 (28.3)	7 (13.0)	9 (14.5)	0.155*

Analysis of variance was performed. *Chi-square analysis was performed.

The values are presented as mean ± SD.

POTS, postural orthostatic tachycardia syndrome; OI, orthostatic intolerance; QTc, corrected QT; AFT, autonomic function test; HRDB, heart rate response to deep breathing; QSART, quantitative sudomotor axon reflex test.

### QTc interval and dispersion

**Figure 1:**
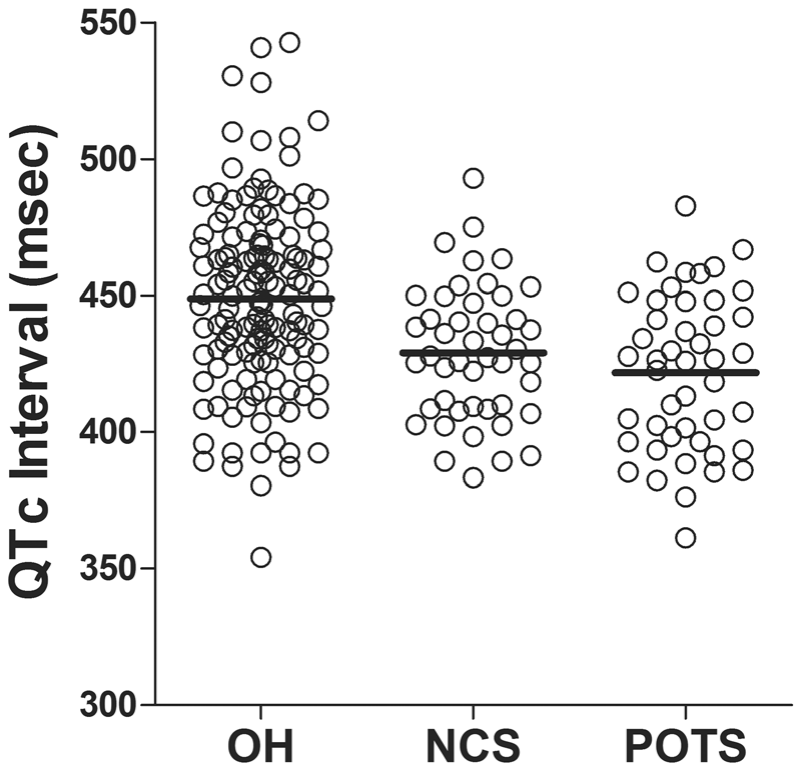
Findings of the corrected QT interval. Patients with orthostatic hypotension (OH) had a more prolonged corrected QT (QTc) interval (448.8±33.6 msec) than patients with neurocardiogenic syncope (NCS) (429.1±24.6 msec, *p* = 0.001) and postural orthostatic tachycardia syndrome (POTS) (421.7±28.6 msec, *p*<0.001). No difference in QTc interval was observed between NCS and POTS patients (*p* = 0.766).

OH patients showed the longest (448.8±33.6 msec), POTS patients showed the shortest (421.7±28.6 msec), and NCS patients showed an intermediate QTc interval (429.1±24.6 msec; *p*<0.001, Figure 1). The Bonferroni post-hoc procedure showed that QTc intervals of the OH group differed significantly from both the NCS (*p* = 0.001) and POTS (*p*<0.001) groups; however, there was no difference in QTc interval between NCS and POTS patients (*p* = 0.766). ANCOVA revealed significant differences in QTc interval by group remained (*p* = 0.003) even after controlling for the age and gender effects.

**Figure 2:**
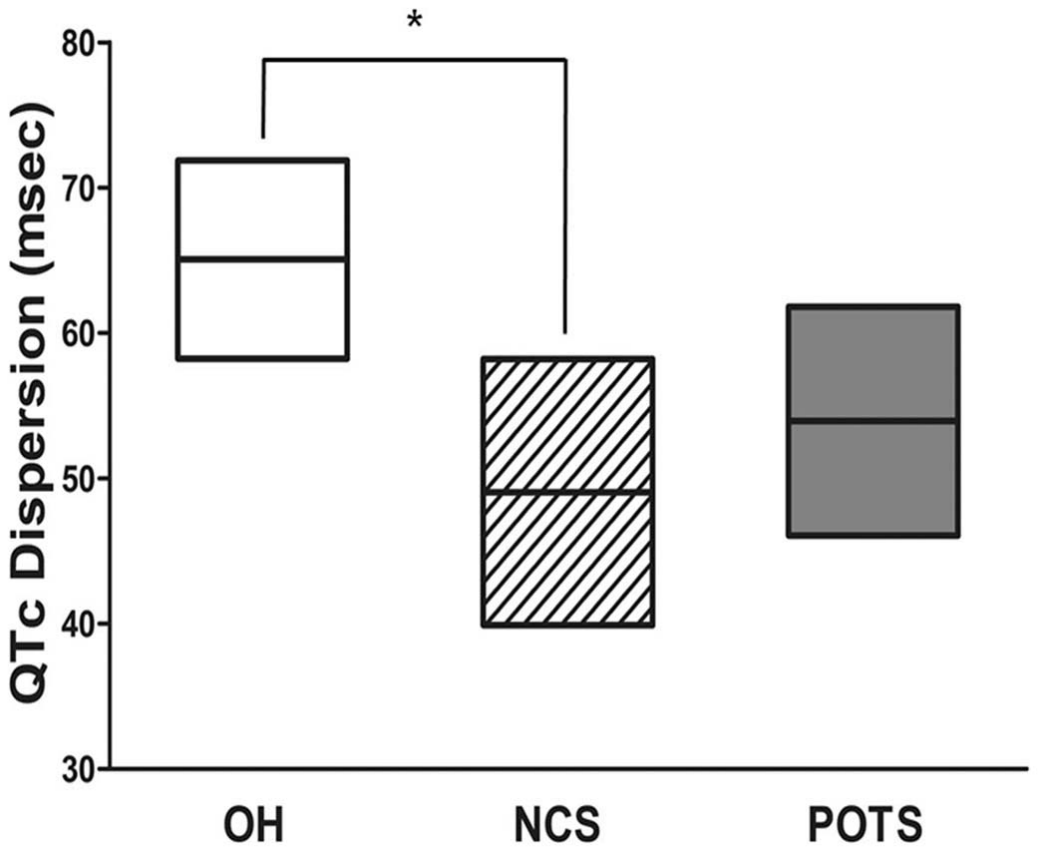
QTc dispersion results. Comparisons of the QTc dispersion showed significant differences between groups (*p* = 0.021). Post-hoc analysis revealed that patients with orthostatic hypotension (OH) had more increased QTc dispersion than patients with neurocardiogenic syncope (NCS) (uncorrected *p* = 0.037, age and gender-corrected *p* = 0.013). The top and bottom borders of the boxes represent the upper and lower limits of the 95% CI range, respectively. The horizontal lines in the middle represent the mean value. Asterisk (*) indicates significant results after correction for post-hoc comparisons.

When analyzing the QTc dispersion, OH patients showed the longest (65.1±32.2 msec), NCS patients showed the shortest (49.0±23.2 msec), and POTS patients showed an intermediate QTc dispersion (53.9±20.7 msec; *p* = 0.021, Figure 2). The Bonferroni post-hoc procedure revealed that QTc dispersion was significantly different only between the OH and NCS groups (*p* = 0.037). ANCOVA also revealed significant differences in QTc dispersion between OH and NCS groups (*p* = 0.013) even after controlling for age and gender effects.

### Autonomic function tests

**Figure 3:**
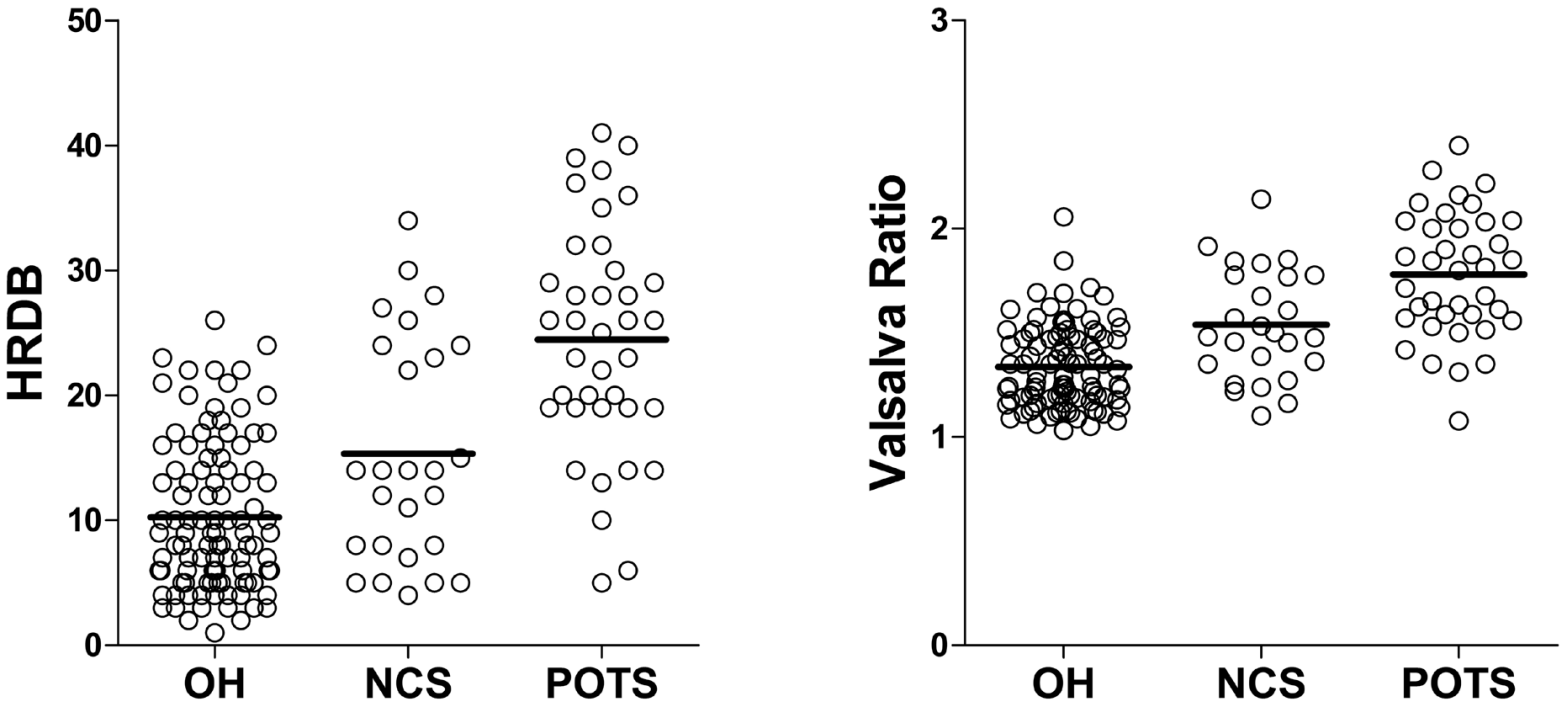
Results of heart rate response to deep breathing and Valsalva ratio. Orthostatic hypotension (OH) patients showed the smallest values in both heart rate response to deep breathing (HRDB; 10.3±6.0 beats/min) and Valsalva ratio (1.34±0.20) and postural orthostatic tachycardia syndrome (POTS) patients showed the largest values in both HRDB (24.5±9.2 beats/min) and Valsalva ratio (1.78±0.30). Neurocardiogenic syncope (NCS) patients showed intermediate levels of HRDB (15.4±9.1 beats/min) and Valsalva ratio (1.54±0.20) compared with OH and POTS patients.

AFT measures and proportions of abnormal results are presented in Table 1. There were significant differences in HRDB and Valsalva ratio between groups. Patients with OH had smaller HRDBs (10.3±6.0 beats/min) than NCS (*p* = 0.006) or POTS (*p*<0.001) patients. In addition, patients with OH had smaller Valsalva ratios (1.3±0.2) than NCS (*p* = 0.001) or POTS (*p*<0.001) patients. Patients with POTS had larger HRDB values (24.5±9.2 beats/min) and Valsalva ratios (1.8±3.0) than OH or NCS (all *p*<0.001) patients. Patients with NCS exhibited intermediate HRDB values (15.4±9.1 beats/min) and Valsalva ratios (1.5±0.3; Figure 3).

**Figure 4:**
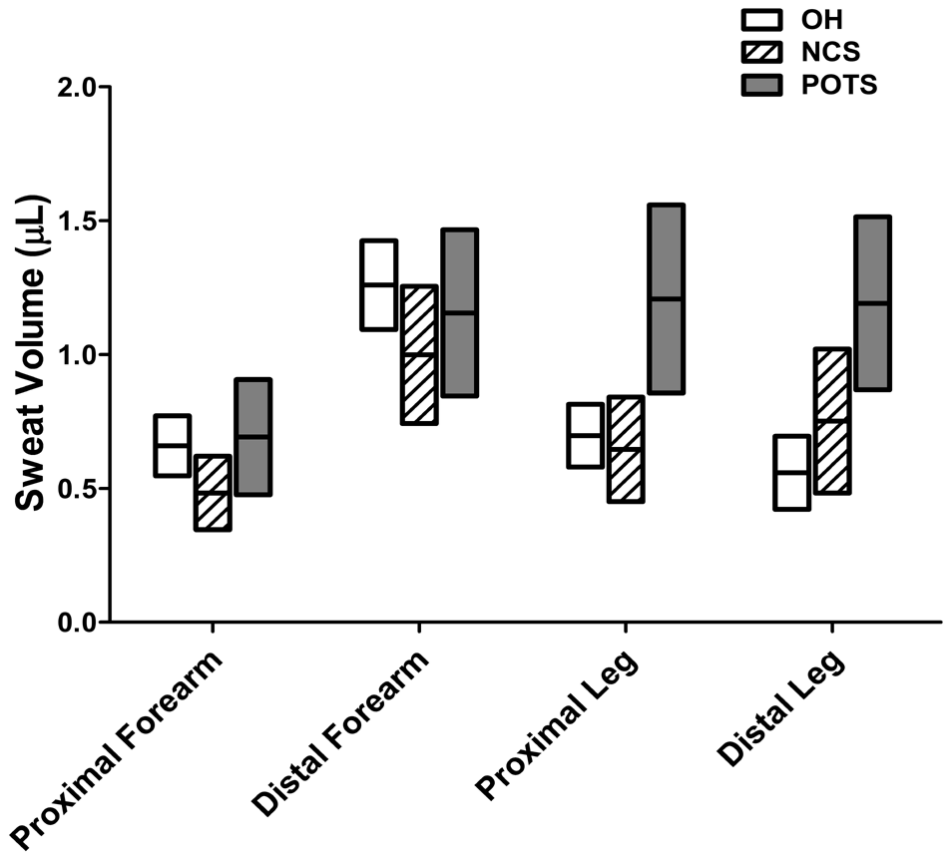
The quantitative sudomotor axon reflex test results according to body site. Postural orthostatic tachycardia syndrome (POTS) patients had greater proximal leg sweat volume (1.21±0.97 µL) than OH (*p* = 0.001) and NCS (*p* = 0.006) patients. In addition, patients with POTS had greater distal leg sweat volume (1.19±0.85 µL) than OH patients (*p*<0.001). No difference in proximal and distal leg sweat volume between OH and NCS patients was observed. No difference in proximal and distal forearm sweat volume was found between groups. The top and bottom borders of the boxes represent the upper and lower limits of the 95% CI range, respectively. The horizontal lines in the middle represent the mean value.

The proportion of abnormal results did not vary between groups in the QSART (*p*>0.05). In a subanalysis of the QSART based on recording site, patients with POTS had a greater sweat volume of the proximal leg (1.21±0.97 µL) than OH (*p* = 0.001) or NCS (*p* = 0.006) patients. In addition, patients with POTS had a greater sweat volume of the distal leg (1.19±0.85 µL) than OH patients (*p*<0.001; Figure 4). There were no QSART differences between OH patients and NCS patients (all *p*>0.05).

### Relationship between ECG measurements and autonomic function tests

**Figure 5:**
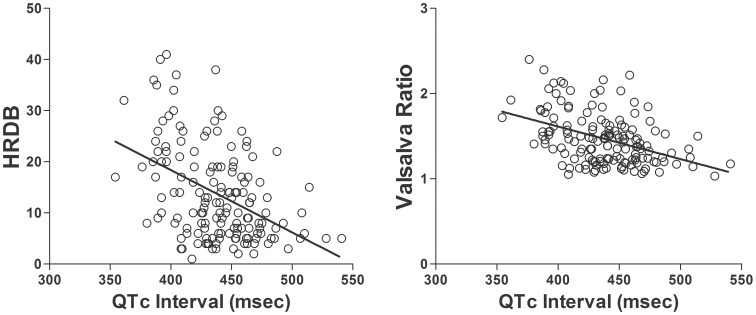
Correlation of corrected QT interval with heart rate response to deep breathing and Valsalva ratio. The corrected QT (QTc) interval showed negative correlations with HRDB (*r* = −0.443, *p*<0.001, left panel) and Valsalva ratio (*r* = −0.425, *p*<0.001, right panel). In the partial correlation analysis controlling for age, associations between QTc interval and HRDB (*r* = −0.364, *p*<0.001), and QTc interval and Valsalva ratio (*r* = −0.336, *p*<0.001) remained statistically significant.

The QTc interval was positively correlated with age (*r* = 0.297, *p*<0.001), and negatively correlated with HRDB (*r* = −0.443, *p*<0.001) and the Valsalva ratio (*r* = −0.425, *p*<0.001) (Figure 5). In the QSART subanalysis, shorter QTc intervals were associated with greater sweat volumes recorded at the proximal (*r* = −0.164, *p* = 0.042) and distal leg (*r* = −0.252, *p* = 0.008). When controlling for age in the partial correlation analysis, associations between QTc interval and HRDB (*r* = −0.364, *p*<0.001), QTc interval and the Valsalva ratio (*r* = −0.336, *p*<0.001), and QTc interval and sweat volume recorded at the distal leg (*r* = −0.172, *p* = 0.034) remained statistically significant. The QTc dispersion was not correlated with AFT measures.

## Discussion

This study utilized a combined AFT-ECG analysis to differentiate patterns of autonomic dysfunction between OH, NCS, and POTS patients. We found that OH patients had greater autonomic dysfunction in several domains, including the cardiovagal function. Patients with OH exhibited the most prolonged QTc interval and dispersion of the three groups and POTS patients had greater leg sweat volume. In addition, the QTc interval was negatively correlated with the Valsalva ratio and HRDB.

The fundamental pathophysiological mechanism underlying OH may be the impairment of systemic vascular resistance during standing, resulting in pooling of blood distally, leading to hypotension [3]. The term “*idiopathic OH*” was first used to describe patients who had OH, regardless of other conditions [28]; however, various autonomic dysfunctions could be combined in patients with OH, including bowel, sudomotor, thermoregulatory, and sexual [4]. Moreover, somatic symptoms such as parkinsonism, have been reported in patients with OH. Therefore, OH patients are currently classified based on combined symptoms into pure autonomic failure, Parkinson's disease with autonomic failure, and multiple system atrophy [25]. Our finding of autonomic dysfunction in both cardiovagal and sudomotor sympathetic domains in OH patients support the previous hypothesis that OH may be one symptom of degeneration of the autonomic nervous system. Because a relatively small proportion of OH patients in our study had comorbid Parkinson's disease (22.6%) or multiple system atrophy (7.5%), we speculate this group is mainly composed of patients with pure autonomic failure. The similarity of clinical characteristics between the OH patients included in our study (mean age, 67.1 years; male-to-female ratio, 1.8∶1; Table 1) and patients with autonomic failure included in previous studies [4], [29] further support this hypothesis. Our finding of prolonged QTc interval, increased QTc dispersion, and widespread impairment in autonomic function domains in OH patients could be interpreted as sympathetic and parasympathetic dysfunctions, which are characteristic features of pure autonomic failure.

The pathophysiological mechanism of NCS remains unclear; however, the paradoxical compensatory reflex known as ‘ventricular theory’ has been widely accepted as the most probable mechanism [30]–[32]. Although distinct pathophysiological interim pathway mechanisms have been suggested, various hypotheses predict a relative increase in parasympathetic activity [32]. Given the reciprocal interaction between sympathetic and parasympathetic control of cardiac functions [33], [34], NCS may be caused by altered sympathovagal balance in response to triggers, rather than inherent autonomic impairment in each domain. Our finding of a relatively lower proportion of abnormality in AFTs supports this speculation. In addition, our findings of the shortest QTc dispersion (49.0±23.2 msec) within the upper limit of normal range (50 msec) in NCS patients further support our hypothesis [35].

Similar to NCS patients, POTS patients showed a small proportion of abnormality in AFTs. These findings are consistent with previous findings that autonomic functions were preserved in a majority of POTS patients [36]. Various hypotheses, such as hypovolemia [37], venous pooling [38], hyperadrenergic states [39]–[41], and restricted adrenergic neuropathies [42] have been suggested to be possible mechanisms underlying POTS. In a previous study using QSART, patients with neuropathic POTS showed loss of sweating in the lower limb [43]. Our study showed increased lower limb sweat volume in POTS patients, suggesting increased sympathetic activity. Since heterogeneous conditions comprise POTS [39], [44], differences in population characteristics might account for the inconsistent QSART findings across studies. Our QSART findings showing a relative increase in sweat volume in the lower limb support the concept that increased sympathetic activity may be a pathophysiological mechanism of POTS.

QTc interval is an ECG measure of the duration between ventricular depolarization and repolarization, which is the ventricular refractory period indicating the shifting of sympathetic to parasympathetic activity [11]. A QTc prolongation of more than 440 msec is a predictor of increased risk of malignant ventricular arrhythmias and sudden death [11]. QTc interval may be prolonged in patients with an altered balance between sympathetic and parasympathetic activities [45]. In particular, prolonged QTc interval is associated with delayed ventricular repolarization mediated by a decline in parasympathetic activity. Therefore, the predictive value of prolonged QTc in sudden death can be explained as ventricular electrical instability associated with high sympathetic activity combined with a decline in parasympathetic activity [45]. Our findings of a negative relationship between QTc interval and measures of HRDB and Valsalva ratio further support this explanation.

Additionally, increased QTc dispersion has been used as a biomarker for predicting life-threatening arrhythmogenic conditions, which has similar clinical implications to prolonged QTc interval [20]. However, important considerations with respect to the differences between QTc interval and QTc dispersion exist. For example, when sympathetic denervation involves the entire myocardium, ventricular repolarization duration would be homogenously prolonged and result in prolonged QTc interval without increased QTc dispersion [12]. The QTc dispersion reflects spatial heterogeneity of QTc interval among the 12 ECG leads depending on differences in repolarization duration of myocardium [46]. Therefore, QTc dispersion inherently tends to be determined by the distribution heterogeneity of a damaged autonomic nervous system within the myocardium, rather than overall sympathovagal modulation. Indeed, increased QTc dispersion has been reported mostly in conditions involving structural changes of the myocardium, such as ventricular hypertrophy, regional (vascular territorial) myocardial damage after myocardial infarction, and congestive heart failure [47]. Conversely, QTc interval could be influenced more by overall cardiac autonomic modulation than QTc dispersion [12]. Our findings indicating that AFT measures correlated only with QTc interval and not with QTc dispersion further support this concept.

QTc interval can be a robust measure for screening OH patients with autonomic failure, especially those presenting with cardiovagal dysfunction in terms of arrhythmogenic condition [20], and is cost-effective and a simple measurement method that does not require patient compliance [12], [48].

There are several possible limitations to consider. Firstly, OI is composed of highly heterogeneous etiological conditions. Therefore, these comorbid conditions may comprise a set of unknown confounding factors affecting AFTs and QTc intervals, despite statistically controlling for known confounding effects. Our findings should be interpreted as reflecting autonomic dysfunction and altered sympathovagal balance in each category of OI, regardless of etiological conditions. Secondly, a significant difference in age existed between groups. Therefore, despite controlling for age, we could not entirely eliminate the effect of age on AFTs or QTc interval. Further prospective investigation of similarly aged populations in all 3 groups is needed. Thirdly, we did not include control subjects in the current study. Therefore, whether our findings of negative correlation between cardiovagal domain in AFTs and QTc interval could be interpreted as general findings or only an OI-specific feature remains unclear. Further study investigating a relationship between AFTs and QTc interval/dispersion in normal healthy subjects would provide useful information for understanding the value of QTc interval/dispersion in the autonomic nervous system. However, the aim of this study was to analyze reliability of QTc interval to investigate differences in patterns of altered sympathovagal balance among the 3 disease groups (OH, NCS and POTS) rather than comparing the QTc interval between patients with OI and normal healthy subjects. Given the aim of study, our study design was reasonable to suggest that QTc interval could be a surrogate biomarker differentiating patterns of altered sympathovagal balance in patients with OI, even though control group was not included to analysis. Therefore, we think that the lack of control group is not a critical limitation in this study. Lastly, our ECG and AFT data were obtained during the resting state. Acquisition of ECG and AFT data during symptomatic periods may provide valuable information regarding any differences in sympathovagal balance between OI groups. Despite these limitations, identification of distinct differences between AFTs and QTc interval by group in each category of OI can be considered relevant in this study. Moreover, our study showed that QTc interval was negatively correlated with HRDB and Valsalva ratio, suggesting the utility of QTc interval as a screening tool for patients with cardiovagal dysfunction.

In conclusion, using combined composite AFTs and the QTc interval, we found distinct patterns of AFT measures in each category of OI. Due to the negative correlations between QTc interval and AFT values representing cardiovagal function, we suggest that prolonged QTc interval can be considered a biomarker for screening alterations in sympathovagal balance, especially cardiovagal dysfunction in OH.
