# Correction: Utility of Corrected QT Interval in Orthostatic Intolerance

**DOI:** 10.1371/journal.pone.0118166

**Published:** 2015-02-06

**Authors:** The PLOS ONE Staff

There is an error in affiliation 3 for author Ki-Jong Park. Affiliation 3 should be: Department of Neurology, Institute of Health Science, Gyeongsang National University School of Medicine, Jinju, Korea.
